# Inorganic Dual-Gate ZnO–Te Anti-Ambipolar Switches for Electrically Programmable Window Detection and Multi-Level Voltage Classification

**DOI:** 10.3390/nano16211418

**Published:** 2026-11-04

**Authors:** Byeong Gwan Kim, Jae Hyeon Jun, Jun-Hyeok Choi, Hyeon-Seok Shin, Jung-Hun Jo, Chan Bin Lee, Jun Young Hong, Jae Min Jo, Byoung Hun Lee

**Affiliations:** 1 Graduate School of Semiconductor Technology, Pohang University of Science and Technology, Cheongam-ro 77, Nam-gu, Pohang 37673, Republic of Korea; kimbg2613@postech.ac.kr (B.G.K.); jun6293@postech.ac.kr (J.-H.C.); junghun@postech.ac.kr (J.-H.J.); color0522@postech.ac.kr (J.Y.H.); jjm0432@postech.ac.kr (J.M.J.); 2 National Institute for Nanomaterials Technology, Pohang University of Science and Technology, Cheongam-ro 77, Nam-gu, Pohang 37673, Republic of Korea; jhjun@postech.ac.kr; 3 Department of Electrical Engineering, Pohang University of Science and Technology, Cheongam-ro 77, Nam-gu, Pohang 37673, Republic of Korea; tjr4117@postech.ac.kr (H.-S.S.); cksqls222@postech.ac.kr (C.B.L.)

**Keywords:** heterojunction, anti-ambipolar switch, dual-gate, window comparator, voltage classification, CMOS-compatible system

## Abstract

The anti-ambipolar switch (AAS) produces a bell-shaped (Λ) transfer characteristic that embeds two threshold edges within a single device; however, this feature has been utilized mainly for discrete logic and periodic signals, rather than for discriminating a continuous input against defined voltage thresholds. Herein, a dual-gate ZnO–Te AAS (DGAAS), combined with a load transistor into an AAS cell, functions as an electrically programmable window detector. The bounded conduction interval of its Λ-curve defines the detection window, and the top-gate bias relocates this window without changes to the circuit topology. Fabricated at ≤150 °C, the device translates its peak by 0.95 V per volt of top-gate bias, with a device-to-device standard deviation of 29.4 mV across 30 devices. A six-transistor window detector, comprising the AAS cell and an output buffer, replaces the two-comparator-and-AND structure of a conventional CMOS design, reducing the transistor count by 75–77%, and circuit simulations based on the measured cell characteristics show that three cells map a continuous analog input to a 2-bit digital output. These results indicate that the ZnO–Te DGAAS serves as a compact primitive for voltage-domain signal discrimination, connecting analog sensing and digital readout within a back-end-of-line (BEOL)-compatible process.

## 1. Introduction

As the physical scaling of silicon CMOS approaches its fundamental limits, traditional transistor miniaturization is increasingly insufficient to meet the escalating demands for area and power efficiency [1,2]. This bottleneck has catalyzed a paradigm shift toward novel device primitives that exhibit transfer characteristics beyond the monotonic response of conventional field-effect transistors (FETs) [3,4]. By enabling a single device to execute complex functions that typically require multiple interconnected transistors, this approach circumvents the traditional scaling constraint—where functional complexity is strictly dictated by device count—thereby offering a highly promising pathway for ultra-compact circuit architectures [5].

Among these primitives, the anti-ambipolar switch (AAS) has emerged as a particularly compelling candidate [6]. By integrating n-type and p-type channels in series across a heterojunction with a shared gate, conduction in the AAS occurs exclusively when both channels are simultaneously activated. This synergistic operation yields a distinctive bell-shaped (Λ) drain current profile that peaks at an intermediate gate voltage and diminishes sharply on either side. Such non-monotonic behavior inherently facilitates the compact implementation of advanced computational elements, including multi-valued logic [7], frequency multiplication [8], and neuromorphic computing architectures [9].

While AAS devices have been demonstrated using diverse materials, including two-dimensional transition metal dichalcogenides (TMDs) [10,11] and organic semiconductors [12,13], integration challenges remain. TMD-based AAS devices typically depend on mechanical exfoliation, which limits wafer-scale scalability [10,11]. Organic counterparts exhibit challenges with channel thickness control and long-term stability, restricting their application in integrated circuits [14]. Furthermore, the operational flexibility of single-gate AAS devices is constrained because the peak position of the Λ-curve is determined by the intrinsic threshold voltages of the constituent channels. Although a dual-gate architecture has been proposed to electrically tune this peak position via an auxiliary gate, implementations have largely been restricted to organic or exfoliated van der Waals channels, which retain the aforementioned material limitations [15,16]. Consequently, developing inorganic channel materials compatible with large-area, uniform processing is necessary for the circuit-level deployment of dual-gate AAS devices.

To overcome these material limitations, we employ an inorganic heterojunction comprising ZnO and Te. Unlike the TMD and organic channels discussed above, oxides such as ZnO provide reliable n-type channels, whereas high-mobility p-type oxides remain limited owing to the strong localization of holes in metal oxides [17], motivating the use of a non-oxide p-type counterpart. The n-type ZnO channel is deposited via atomic layer deposition (ALD), providing a wide bandgap and suppressed off-currents [18,19,20]. The Te layer serves as an intrinsic p-type channel with high carrier mobility, depositable via low-temperature sputtering and ALD [21,22]. The fabrication process is executed at a wafer scale with a low thermal budget (≤150 °C), ensuring compatibility with back-end-of-line (BEOL) integration. Previous studies on this material system have demonstrated its applicability for compact logic; for instance, a binary-to-ternary converter utilizing a cross-coupled ZnO–Te AAS cell reduced the device count by approximately 95% and area by 97%, while a frequency quadrupler utilizing double-peak negative differential transconductance (NDT) reduced the device count by 64–75% [23,24]. However, both demonstrations relied on single-gate devices whose Λ-curves are fixed at fabrication, so that each new operating point requires a redesigned device; electrical programming of the peak position has not yet been demonstrated in this material system.

The Λ-shaped transfer characteristics can be utilized beyond logic and frequency domains. Current conduction restricted to a bounded input voltage interval aligns with the operation of a window comparator, which determines whether an analog signal resides within a predefined voltage band. Such band-limited decisions underpin analog signal supervision across power management and industrial control, where they are conventionally implemented with dedicated comparator circuits [25,26]. Because conventional FETs with monotonic transfer characteristics cannot perform this band-discrimination independently, CMOS window comparators require two single-threshold comparators coupled with an AND gate. This structural requirement imposes a hardware minimum that increases with each additional detection threshold. The AAS, however, embeds both the upper and lower threshold edges within the transfer characteristic of a single device. Despite this capability for continuous analog input classification, the AAS has not yet been applied to voltage-domain signal discrimination.

Herein, we demonstrate that the Λ-curve of a ZnO–Te dual-gate AAS (DGAAS) functions as an electrically programmable window detector. By integrating a DGAAS with a load transistor, the bounded conduction interval establishes a detection band, and the top-gate bias relocates this window without topological changes to the circuit. This peak translation occurs at a near-unity rate per volt of top-gate bias, maintaining a constant peak current and shifting the detection band across the input range. Using a single AAS cell, the proposed window detector replaces the two-comparator-and-AND structure of conventional CMOS equivalents, resulting in a 75–77% reduction in transistor count and an over 50% decrease in the projected power-delay product. In a multi-level configuration evaluated by circuit simulation, three DGAAS cells map a continuous analog input to a 2-bit digital output. This architecture reduces the core device count by approximately half compared to a conventional comparator-based flash analog-to-digital converter (ADC) for 2- to 4-bit resolutions. These results indicate that the ZnO–Te DGAAS serves as a compact primitive for voltage-domain signal discrimination, connecting analog sensing and digital readout within a wafer-scale, BEOL-compatible process.

## 2. Materials and Methods

Initially, 300 nm SiO_2_/p^++^Si substrates were cleaned sequentially with acetone, methanol, and deionized (DI) water for 5 min each in an ultrasonic bath. The SiO_2_ layer was patterned and dry-etched to a depth of 80 nm using inductively coupled plasma reactive-ion etching (ICP-RIE) with Ar and CF_4_ gases to form a trench for the buried gate structure. Ti/Ni (10/70 nm) was deposited into the trench by electron-beam evaporation and patterned via a lift-off process. A 10 nm Al_2_O_3_ bottom gate dielectric was deposited by atomic layer deposition (ALD) at 150 °C. A 6 nm ZnO layer was deposited by ALD at 120 °C and subsequently patterned by wet etching with a diluted HCl solution (5 mL HCl in 300 mL DI water using a wet etch station (POSTECH Center for Semiconductor Technology Convergence supported by Korea Basic Science Institute, [NFEC-2025-02-304149])). A 4 nm Te layer was deposited by DC sputtering and defined via a lift-off process. Ni source/drain electrodes (60 nm) were deposited by electron-beam evaporation and patterned via a lift-off process. A 10 nm Al_2_O_3_ top gate dielectric was deposited by ALD at 150 °C. Finally, a Ni top gate electrode (60 nm) was deposited by electron-beam evaporation and patterned via a lift-off process.

The electrical characteristics of the fabricated DGAAS devices were measured using a semiconductor parameter analyzer (Keithley 4200A-SCS, Tektronix, Beaverton, OR, USA) in ambient air at 25 °C. Static drain current–gate voltage (I_D_–V_G_) curves were obtained as a function of the bottom gate voltage (V_BG_) at various top gate voltages (V_TG_) to characterize the peak position tunability. The window detector operation of the AAS cell was assessed by measuring the output voltage (V_out_) while sweeping the input voltage (V_in_) under different V_TG_ conditions. 

Circuit-level simulations were performed in Synopsys HSPICE (version P-2019.06-SP1-1; Synopsys, Sunnyvale, CA, USA). The AAS cell was represented by a compact model calibrated to the measured DGAAS and AAS-cell transfer characteristics. To project the performance of scaled devices, the channel of the fabricated devices (L/W = 60/40 µm) was scaled down to 600/400 nm. The CMOS baselines were implemented using the 65 nm Predictive Technology Model (PTM). Device-to-device variation was evaluated by Monte Carlo simulation of the transistor V_th_ mismatch. The time-domain V_out_ responses of the three cells were simulated in HSPICE using their measured DC characteristics, and the resulting digital outputs were obtained by applying these waveforms to an OR-gate encoder. 

## 3. Results and Discussions

**Figure 1:**
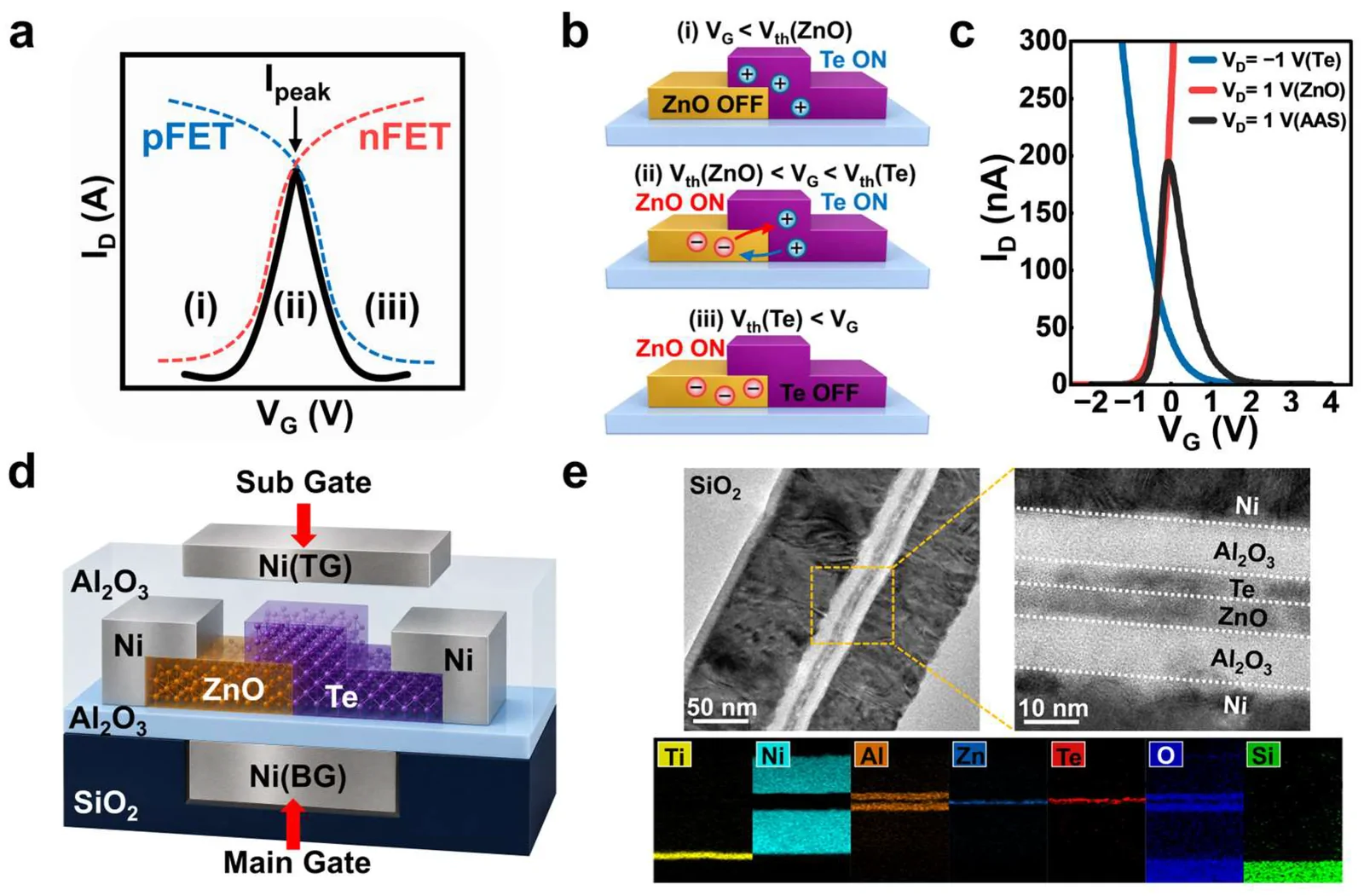
Device structure, carrier transport, and dual-gate design for peak-position control in a ZnO–Te anti-ambipolar switch (AAS). (**a**) Schematic of the I_D_–V_G_ curve of an AAS, where (i)–(iii) denote the three gate-voltage regions. (**b**) Operating mechanism of the AAS. (**c**) Measured I_D_–V_G_ curves of the n-type ZnO field-effect transistor (FET), p-type Te FET, and ZnO–Te AAS. (**d**) Schematic of the dual-gate AAS (DGAAS). (**e**) Cross-sectional high-resolution transmission electron microscopy (HR-TEM) image and energy-dispersive X-ray spectroscopy (EDS) mapping of the DGAAS for Ti, Ni, Al, Zn, Te, O, and Si.

Figure 1a depicts the schematic transfer characteristic (I_D_–V_G_) of the AAS, showing a bell-shaped peak compared to the monotonic on/off switching of conventional field-effect transistors (FETs). This profile arises from the series combination of an n-type and a p-type FET, where current flows when both channels are simultaneously activated. The mechanism is illustrated in Figure 1b across three gate-voltage regions. In region (i), where V_G_ < V_th_ (ZnO), the ZnO channel is off while the Te channel is on, blocking conduction through the series connection. In region (ii), V_th_ (ZnO) < V_G_ < V_th_ (Te), both channels are on, resulting in the peak current. In region (iii), V_G_ > V_th_ (Te), the n-type ZnO channel is on while the p-type Te channel is off, suppressing the total current. Figure 1c shows the measured transfer curves of the individual n-type ZnO FET, the p-type Te FET, and the ZnO–Te AAS. Each unipolar FET exhibits a monotonic I_D_–V_G_ response, while their series combination produces the Λ-shaped curve, indicating that the bell-shaped conduction originates from the overlap of the two complementary channels.

Figure 1d presents the schematic of the dual-gate AAS (DGAAS), which includes a top gate above the conventional AAS for peak-position control. The device consists of a bottom gate and a top gate, separated from the ZnO–Te heterojunction channel by Al_2_O_3_ gate dielectrics of nominally equal thickness, designed to provide comparable electrostatic coupling from the two gates. The bottom gate sets the primary operating point, while the top gate independently translates the peak position. The detailed wafer-scale fabrication process is illustrated in Supplementary Figure S1, showing the sequential deposition and patterning steps performed at a low thermal budget (≤150 °C), ensuring compatibility with back-end-of-line (BEOL) integration. The thicknesses of ZnO (6 nm) and Te (4 nm) were chosen to ensure film continuity and stability while suppressing the off-current, which increases with thickness owing to bulk conduction in ZnO and the narrowing bandgap of Te [27,28]. The 10 nm Al_2_O_3_ dielectrics enable low-voltage gate control while keeping the gate leakage at the pA level (Supplementary Figure S2). Figure 1e shows a cross-sectional high-resolution transmission electron microscopy (HR-TEM) image of the ZnO–Te overlap region and the corresponding energy-dispersive X-ray spectroscopy (EDS) elemental mapping for Ti, Ni, Al, Zn, Te, O, and Si. The image shows the vertically stacked gate–dielectric–ZnO–Te structure, and the elemental maps indicate that the ZnO and Te layers form a heterojunction with sharp interfaces.

**Figure 2:**
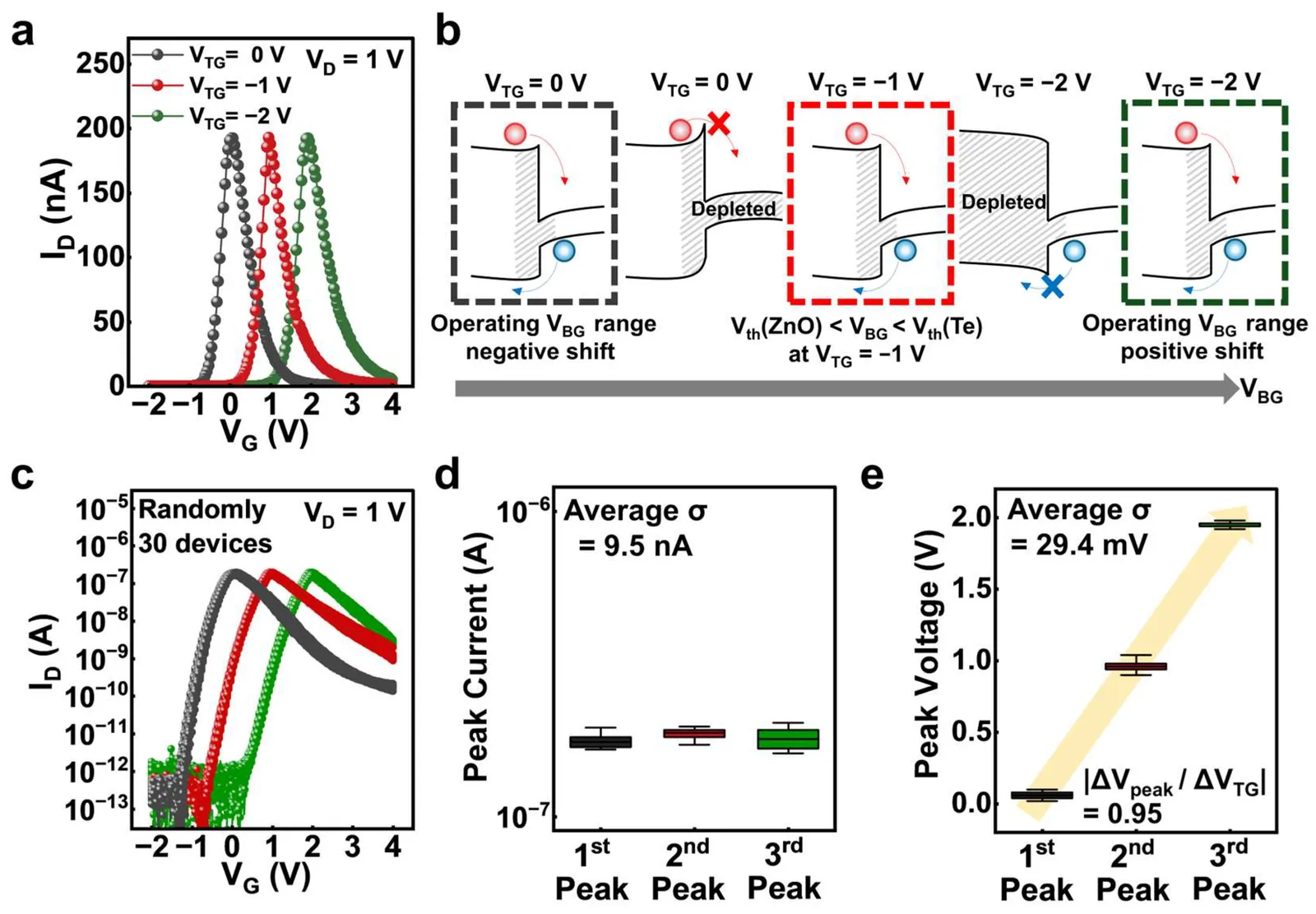
Peak-position tuning and device-to-device uniformity of the DGAAS. (**a**) I_D_–V_G_ curves at V_TG_ = 0, −1, and −2 V (V_D_ = 1 V), showing peak translation with preserved peak current. (**b**) Corresponding band diagrams. Boxed panels mark the operating states where both channels conduct. (**c**) Overlaid I_D_–V_G_ curves of 30 devices per V_TG_ condition, plotted on a logarithmic scale. (**d**) Box plots of the peak current for the 1st, 2nd, and 3rd peaks, with an average device-to-device standard deviation of σ = 9.5 nA. (**e**) Box plots of the peak voltage for the 1st, 2nd, and 3rd peaks, with an average slope of ΔV_peak_/ΔV_TG_ = 0.95 V/V and an average device-to-device standard deviation of σ = 29.4 mV.

Figure 2a shows the I_D_–V_G_ curves of the DGAAS measured under varying top gate voltages (V_TG_), exhibiting peak translation with a constant peak magnitude. The peak is located at approximately 0, 1, and 2 V at V_TG_ = 0, −1, and −2 V (V_D_ = 1 V), respectively, yielding a near-unity translation per volt of V_TG_ modulation. This near-unity ratio corresponds to the comparable electrostatic coupling of the top and bottom gates to the ZnO–Te heterojunction channel, resulting from the symmetric Al_2_O_3_ dielectric thicknesses of the dual-gate stack. The individual ZnO and Te FET characteristics indicate that this translation results from the top-gate modulation of both channel thresholds (Supplementary Figure S2). Figure 2b illustrates the band diagram for each V_TG_ condition in Figure 2a. The boxed panels indicate the operating states where both the ZnO and Te channels conduct simultaneously. As V_TG_ becomes more negative, the threshold voltages of both channels increase, and the operating range shifts to higher V_BG_ values. The unboxed panels show the channel off states at the boundaries of this operating range, where the Te channel (high V_BG_) or the ZnO channel (low V_BG_) is depleted.

For integration into multi-cell circuits, peak parameters must be reproducible across devices, as device-to-device variation affects the precision of threshold-based operations. To evaluate uniformity, the electrical performance of 30 randomly sampled DGAAS devices was analyzed at each of the three top-gate conditions at V_D_ = 1 V. Figure 2c shows the overlaid I_D_–V_G_ curves on a logarithmic scale, with peak parameters summarized in the box plots for peak current (Figure 2d) and peak voltage (Figure 2e). The current amplitude remains at approximately 200 nA across all three V_TG_ conditions, indicating that the top gate modulates the peak position without altering the current magnitude. The box plot yields an average ΔV_peak_/ΔV_TG_ of 0.95 V/V, consistent with the near-unity translation of the peak position. For each condition, the device-to-device standard deviations of the peak current and peak voltage across the 30 devices were 8.6, 7.1, and 12.9 nA and 29.8, 31.2, and 27.2 mV for V_TG_ = 0, −1, and −2 V, respectively, with means of 9.5 nA and 29.4 mV. Because the detection edges of the AAS-based window detector are anchored to this Λ-curve, their positional uncertainty depends on this peak-position variation. Beyond the device-to-device uniformity, the DGAAS was characterized over 28 days of repeated measurements in ambient air, with the device stored in vacuum between measurements; the maximum changes in the peak current and peak voltage relative to day 1 were 7.5 nA and 80 mV, respectively (Supplementary Figure S3).

**Figure 3:**
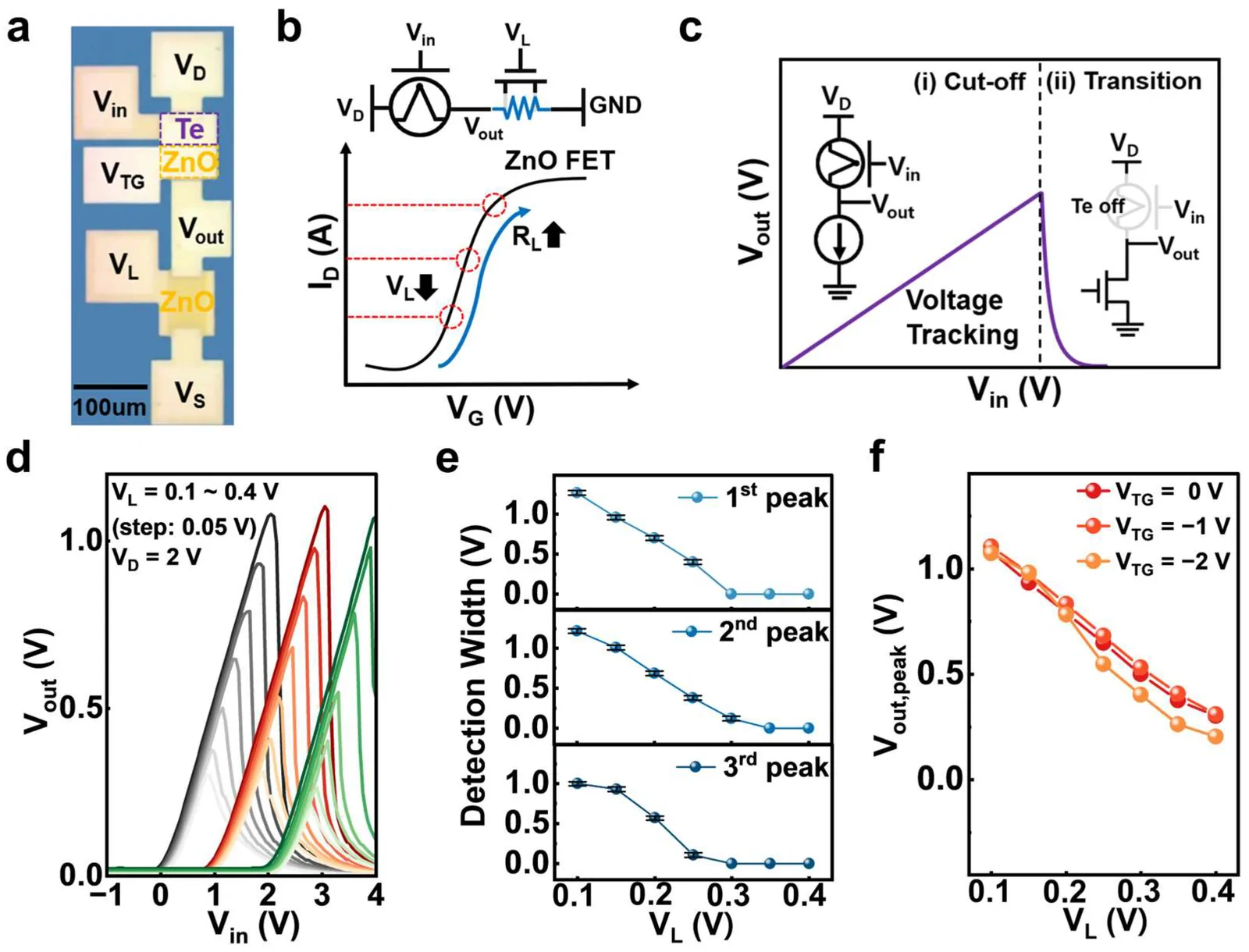
Operating principle and window tunability of the AAS cell. (**a**) Optical microscope image of the AAS cell, comprising a DGAAS in series with a load ZnO FET. (**b**) Operating principle of the AAS cell, illustrating load-resistance modulation by V_L_; the arrows indicate that decreasing V_L_ increases R_L_. (**c**) The two operating regimes, voltage tracking (i) and inverter-like transition (ii). (**d**) Measured V_in_–V_out_ characteristics at V_D_ = 2 V for V_L_ = 0.1–0.4 V (0.05 V steps), at V_TG_ = 0, −1, and −2 V (black, red, and green). (**e**) Detection width, defined as the V_in_ range where V_out_ exceeds 0.5 V, versus V_L_ for the first, second, and third peaks. Error bars denote the peak-position standard deviation σ_AAS_ = 29.4 mV. (**f**) Peak V_out_ as a function of V_L_, following a common trend independent of the peak position.

Figure 3a shows an optical microscope image of the AAS cell, comprising a DGAAS connected in series with a load ZnO FET. The DGAAS is controlled by the input voltage (V_in_) and V_TG_, while the load FET is controlled by its load gate voltage (V_L_). The output voltage (V_out_) is measured at the node between the two devices. Figure 3b illustrates the operating principle. As V_L_ decreases, the load FET enters the subthreshold region, increasing its channel resistance (R_L_) and raising V_out_ through voltage division between R_L_ and the DGAAS resistance. The maximum current of the AAS cell is limited by the increased R_L_, transforming the bell-shaped DGAAS current into a trapezoidal profile with a constant-current plateau over a finite V_in_ range (Supplementary Figure S4a). Within this plateau, the cell operates as a constant-current source, requiring the gate-to-source voltage of the DGAAS (V_in_ − V_out_) to remain approximately constant. Consequently, the DGAAS operates as a source follower, establishing a linear V_in_–V_out_ transfer characteristic (voltage tracking regime). As V_in_ increases beyond the plateau, the Te channel of the DGAAS enters cut-off, the current is suppressed, and V_out_ decreases toward ground (transition regime). Figure 3c summarizes the two operating regimes of the AAS cell: voltage tracking (i) and transition (ii). The end of the current plateau coincides with the V_in_ at which V_out_ reaches its peak, marking the boundary between the two regimes (Supplementary Figure S4b), consistent with the described mechanism.

Figure 3d presents the measured V_in_–V_out_ characteristics at V_D_ = 2 V for V_L_ ranging from 0.1 V to 0.4 V in 0.05 V steps. Three sets of Λ-shaped V_out_ responses are observed at different V_in_ positions, corresponding to programmed V_TG_ = 0, −1, and −2 V (black, red, and green, respectively). As V_L_ decreases, R_L_ increases, raising the V_out_ peak amplitude and widening the detection window. Figure 3e shows the detection width, defined as the V_in_ range over which V_out_ exceeds the 0.5 V logic threshold, as a function of V_L_ for the first, second, and third peaks. The detection width decreases monotonically as V_L_ increases, from approximately 1 V at V_L_ = 0.1 V to zero at V_L_ = 0.4 V. The error bars indicate the device-to-device variation of the detection edges. Because the edges are anchored to the peak position, this variation corresponds to the peak-position standard deviation extracted in Figure 2 (σ_AAS_ = 29.4 mV). Figure 3f plots the peak V_out_ as a function of V_L_ for the three V_TG_ conditions, showing a uniform trend indicating that the output amplitude is determined by V_L_ independent of the V_TG_-programmed position.

**Figure 4:**
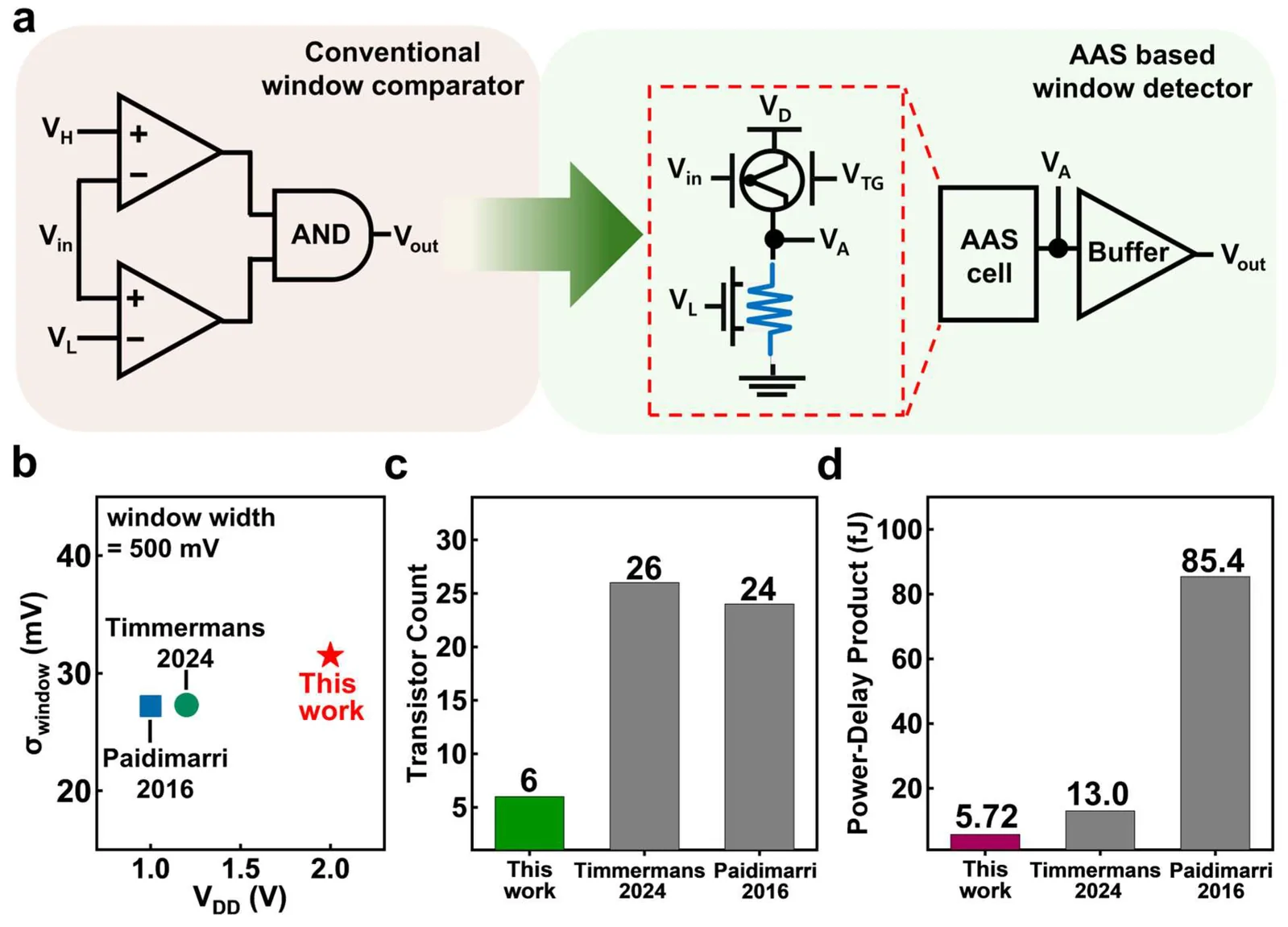
AAS-based window detector versus conventional CMOS window comparators. (**a**) Circuit schematics of the two architectures. (**b**) σ_window_, the device-to-device standard deviation of the detection window width, plotted against supply voltage for the three designs; Timmermans 2024 and Paidimarri 2016 denote the CMOS baselines built from the comparator cores of Refs. [29] and [30], respectively. (**c**) Transistor count of the AAS-based design and the two CMOS baselines. (**d**) Projected power-delay product (PDP) of the sub-micron-scaled AAS-based design and the two CMOS baselines.

Figure 4a presents the circuit schematics of the two architectures. The AAS-based window detector consists of a single AAS cell followed by an output buffer, where the AAS cell performs the window detection through its Λ-shaped transfer characteristic. In contrast, the conventional CMOS window comparator requires two comparators, one for each of the upper and lower thresholds, followed by an AND gate that combines their outputs into a single window decision. The AAS-based architecture replaces the two comparators and the AND gate with a single AAS cell, reducing the transistor count and power consumption. For comparison, the DGAAS and AAS cell were modeled using a compact model calibrated to the measured characteristics (Supplementary Figure S5a,b) and combined with a CMOS buffer. As baselines, two conventional two-comparator-AND window detectors were built from the continuous-time comparator cores of reported CMOS designs, excluding their system-level circuitry [29,30]. Each baseline was simulated with the 65 nm PTM at its reported supply voltage (1.2 and 1.0 V, respectively), and all three circuits were configured for a 500 mV detection window.

Figure 4b plots σ_window_, the device-to-device standard deviation of the detection window width, for the three designs against supply voltage. At their respective operating supply voltages, the AAS-based detector shows σ_window_ = 31.5 mV (V_DD_ = 2 V), while the CMOS baselines show 27.3 mV and 27.25 mV (V_DD_ = 1.2 V and 1 V), indicating comparable variation. For the AAS-based detector, the window uncertainty originates from two independent stages: the AAS cell and the CMOS buffer. The device-level variation of the AAS cell was taken from the measured peak-position uniformity (σ_AAS_ = 29.4 mV, Figure 2), whereas the buffer contribution was estimated from Monte Carlo simulation of its V_th_ mismatch (σ_buffer_, *N* = 200). As the two stages are statistically independent, the total window variance is expressed as σ_window_^2^ = σ_AAS_^2^ + σ_buffer_^2^. The CMOS baseline values were extracted from full-circuit Monte Carlo simulation under the same V_th_-mismatch framework.

Figure 4c shows the transistor count. The AAS-based design uses 6 transistors, while the CMOS baselines use 26 and 24. To assess the performance of the AAS-based detector, the AAS cell was scaled down to sub-micron dimensions while preserving its L/W ratio, and its power-delay product (PDP) was evaluated against the baseline window comparators (Figure 4d). At these scaled dimensions, the AAS-based design yields a projected PDP of 5.72 fJ, compared with 13.0 fJ and 85.4 fJ for the CMOS baselines. To compare the circuits operating at different supply voltages, the PDPs were normalized by V_DD_^2^, yielding 1.43, 9.03, and 85.4 fJ/V^2^, respectively. Although this normalization does not account for the effects of V_DD_ on the delay and static power consumption, it removes the first-order V_DD_^2^ dependence of the switching energy. These reductions in transistor count and PDP are mainly attributed to the architectural compactness of the AAS-based detector. This result indicates that performing the window operation at the device level, rather than through circuit-level signal combination, provides a method for compact, low-power window detection.

**Figure 5:**
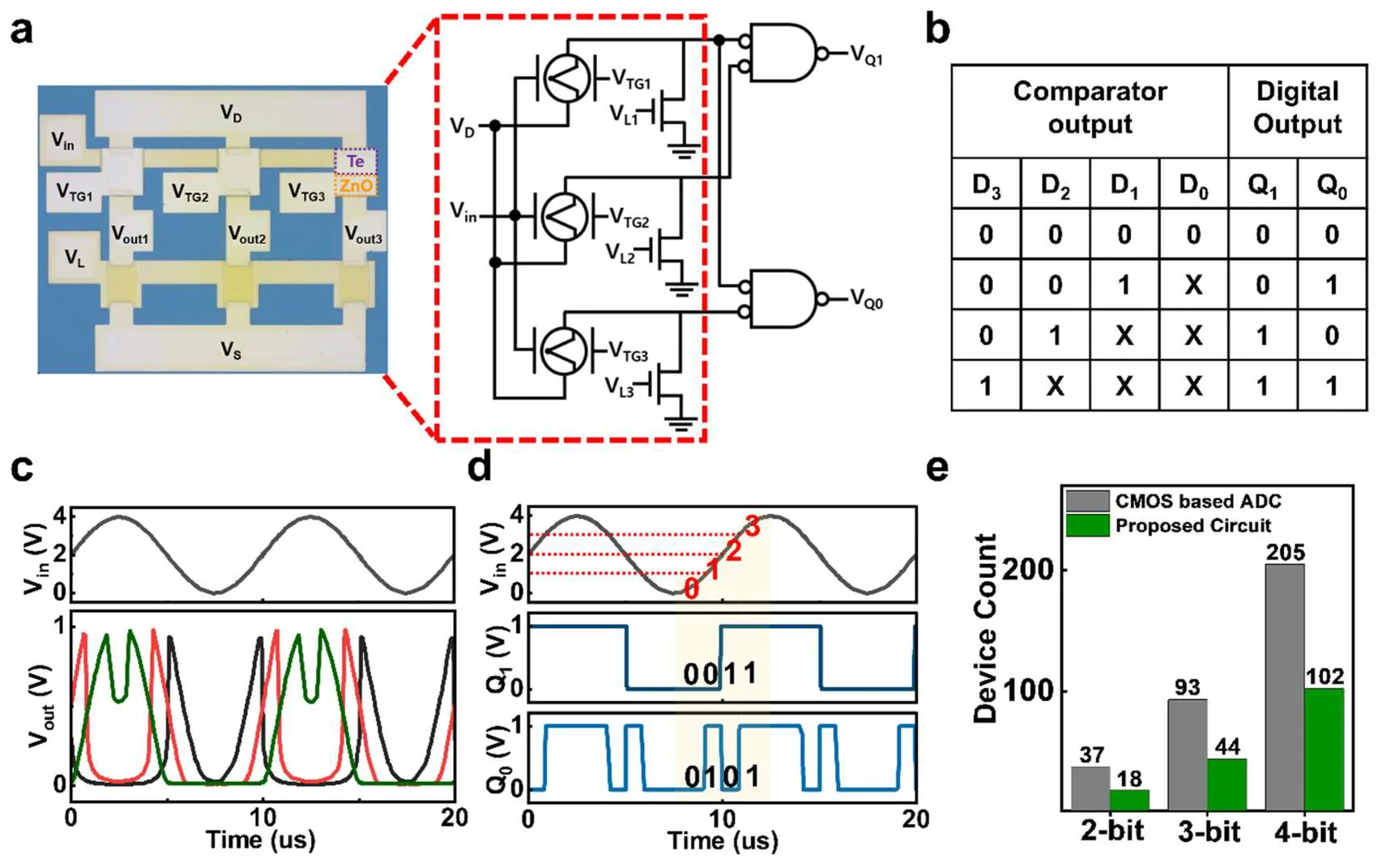
AAS-based multi-level voltage classifier. (**a**) Optical microscope images and circuit schematic showing three AAS cells with top-gate voltages V_TG1_, V_TG2_, V_TG3_ and 2-bit encoder. (**b**) Truth table of the 2-bit encoder, applicable to both the conventional thermometer-coded CMOS flash ADC (X = 1) and the proposed AAS-based classifier (X = 0). (**c**) Simulated time-domain V_out_ responses of the three cells (black, red, and green for V_TG1_, V_TG2_, V_TG3_) to a sinusoidal V_in_. (**d**) Simulated digital outputs Q_1_ and Q_0_ transitioning through 00, 01, 10, and 11 as V_in_ sweeps through the four sensing regions. (**e**) Core transistor count comparison with a conventional CMOS flash ADC for 2-, 3-, and 4-bit discrimination.

Figure 5a shows the proposed AAS-based multi-level voltage classifier, consisting of three AAS cells whose top-gate voltages V_TG1_, V_TG2_, and V_TG3_ are independently programmed to define three distinct sensing levels across the input range of V_in_. Each cell, comprising a DGAAS and a load transistor, exhibits a Λ-shaped transfer characteristic with a peak position determined by V_TG_. Because each cell responds over a specific V_in_ interval, the three cells partition the input range, and their outputs are encoded into the 2-bit binary code Q_1_, Q_0_ through two OR gates. Figure 5b shows the truth table of the encoder, where D_i_ denotes the output of the i-th AAS cell, taken as high when its V_out_ exceeds the 0.5 V logic threshold. The table covers both conventional thermometer coding (X = 1) and the one-hot coding produced here (X = 0), but the two require different logic: thermometer coding needs a priority encoder, whereas the one-hot case reduces to Q_0_ = D_1_ + D_3_ and Q_1_ = D_2_ + D_3_, implemented with two OR gates. This asymmetry is the origin of the transistor-count difference in Figure 5e. Figure 5c shows the time-domain response of the three V_out_ signals to a sinusoidal V_in_, evaluated in HSPICE with the measured characteristics of the fabricated cells, and Figure 5d presents the resulting digital outputs obtained by applying these waveforms directly to the OR-gate encoder in HSPICE. Each V_out_ trace peaks at the V_in_ set by its V_TG_, and the outputs transition through the codes 00, 01, 10, and 11 as V_in_ sweeps through the four regions, confirming the multi-level classification in simulation.

In Figure 5e, the core transistor count of the AAS-based classifier is compared with a conventional CMOS flash ADC employing dynamic latch comparators and a priority encoder (Supplementary Figure S6a,b). The core count includes the comparator or AAS cell elements and the encoder, excluding peripheral circuits common to both implementations, such as input buffers and reference-voltage generators. The AAS-based architecture would use 18, 44, and 102 core transistors for 2-, 3-, and 4-bit discrimination, compared to 37, 93, and 205 for the CMOS counterpart, corresponding to reductions of 51%, 53%, and 50%, respectively. Collectively, the window detection and multi-level classification indicate that the ZnO–Te DGAAS can serve as a compact primitive for voltage-domain signal discrimination, enabling analog sensing and digital readout within a wafer-scale, BEOL-compatible process.

## 4. Conclusions

In summary, a dual-gate anti-ambipolar switch (DGAAS) based on a ZnO–Te heterojunction with a Λ-shaped transfer characteristic, whose peak is electrically tunable via the top-gate voltage, has been demonstrated. The wafer-scale fabrication process exhibits device-to-device uniformity, verified across 30 devices at each top-gate condition, with a low thermal budget (≤150 °C) that ensures compatibility with BEOL integration. Utilizing the bounded conduction interval of the Λ-curve, an AAS-based window detector was evaluated, showing a reduced transistor count and projected power-delay product compared to a conventional CMOS window comparator. Additionally, a multi-level voltage classifier, consisting of three AAS cells coupled to a standard OR-gate encoder, was evaluated by circuit simulation, mapping a continuous analog input to a 2-bit digital output and reducing the core transistor count by approximately 50% compared to a conventional CMOS flash ADC. These results indicate that the ZnO–Te DGAAS serves as a compact primitive for voltage-domain signal discrimination, enabling area-efficient, BEOL-compatible processes that connect analog sensing and digital readout.

## Data Availability

The original contributions presented in this study are included in the article/Supplementary Material. Further inquiries can be directed to the corresponding author.

## Supplementary Materials

The following supporting information can be downloaded at https://www.mdpi.com/article/10.3390/nano16211418/s1, Figure S1: Schematic illustration of the fabrication process flow for the ZnO–Te DGAAS; Figure S2: Electrical characteristics of the individual ZnO and Te FETs; Figure S3: Long-term stability of the Al_2_O_3_ (top oxide)-passivated DGAAS over 28 days; Figure S4: Current plateau and output peak position of the AAS cell; Figure S5: Compact-model fitting to the measured characteristics of the DGAAS and the AAS cell; Figure S6: Baseline CMOS flash ADC composed of dynamic latch comparators and a priority encoder.
