# Supplementary material for: Inorganic Dual-Gate ZnO–Te Anti-Ambipolar Switches for Electrically Programmable Window Detection and Multi-Level Voltage Classification
Source: Nanomaterials (Basel). 2026 Nov 4;16(21):1418. doi: 10.3390/nano16211418 (PMC13765462; doi:10.3390/nano16211418)
Supplement: Supplementary file 1 [file nanomaterials-16-01418-s001.zip › nanomaterials-4643378-supplementary.pdf]

Supporting Information

Inorganic Dual-Gate ZnO–Te Anti-Ambipolar Switches for Electrically Programmable Window Detection and Multi-Level Voltage Classification

Byeong Gwan Kim[1], Jae Hyeon Jun[2], Jun-Hyeok Choi[1], Hyeon-Seok Shin[3], Jung-Hun Jo[1], Chan Bin Lee[3], Jun Young Hong[1], Jae Min Jo[1] and Byoung Hun Lee[1,2,3*]

[1] Graduate School of Semiconductor Technology, Pohang University of Science and Technology, Cheongam-ro 77, Nam-gu, Pohang 37673, Republic of Korea
[2] National Institute for Nanomaterials Technology, Pohang University of Science and Technology, Cheongam-ro 77, Nam-gu, Pohang 37673, Republic of Korea
[3] Department of Electrical Engineering, Pohang University of Science and Technology, Cheongam-ro 77, Nam-gu, Pohang 37673, Republic of Korea
* Correspondence: bhlee1@postech.ac.kr

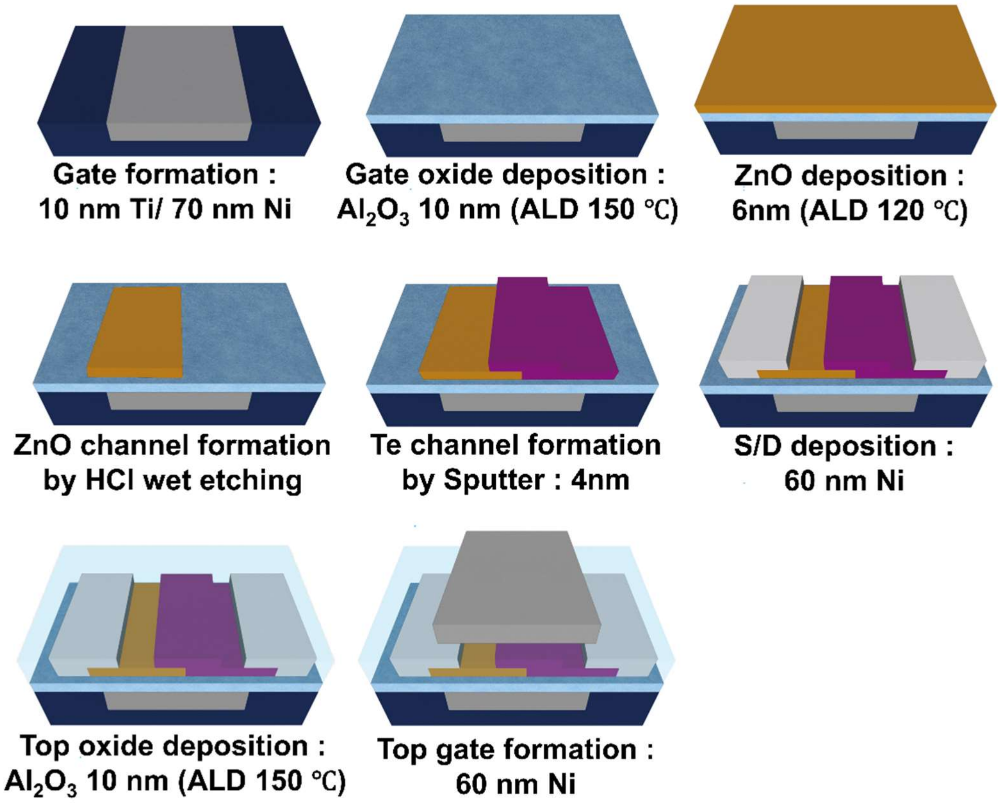

Figure S1. Schematic illustration of the fabrication process flow for the ZnO–Te DGAAS.

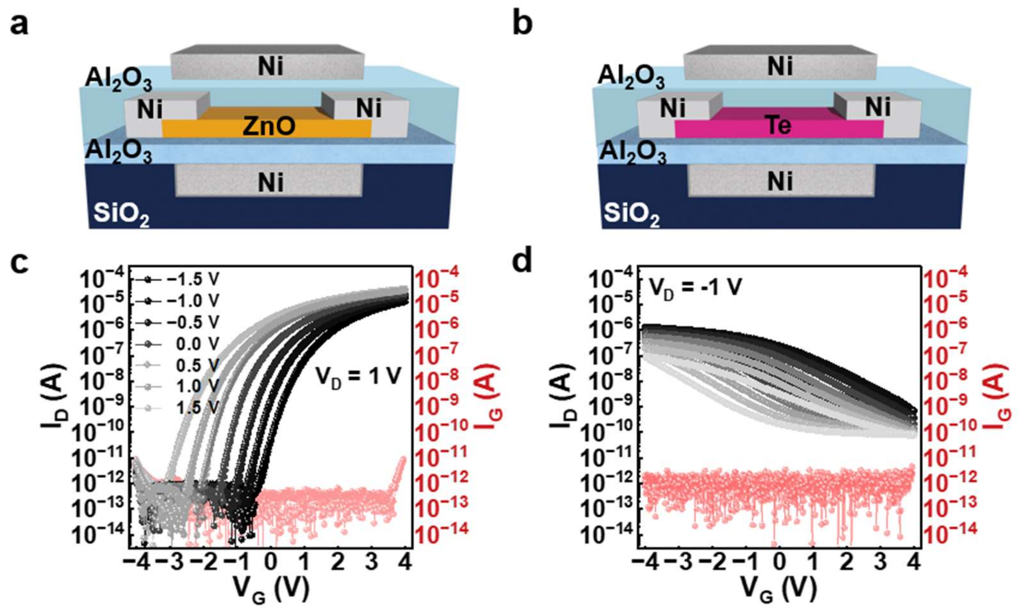

Figure S2. Electrical characteristics of the individual ZnO and Te FETs. Structure of (a) the n-type ZnO FET and (b) the p-type Te FET. Transfer curves (I_D–V_G) of (c) the ZnO FET (V_D = 1 V) and (d) the Te FET (V_D = −1 V). Each transfer curve corresponds to a top-gate voltage (V_{TG}) from −1.5 to 1.5 V in 0.5 V steps, which modulates the threshold voltage.

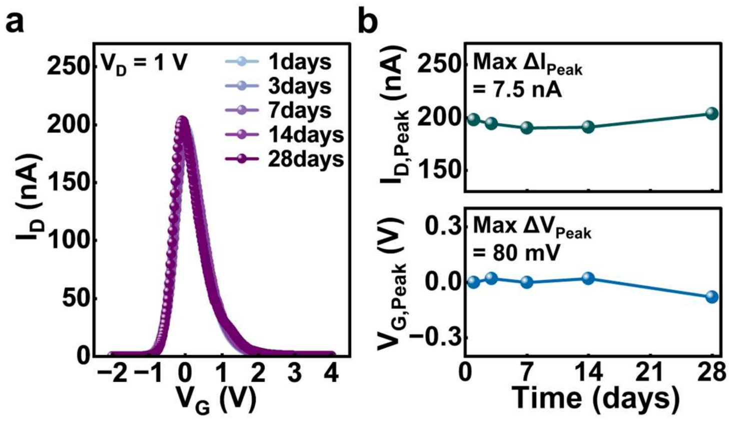

Figure S3. Long-term stability of the Al_2O_3 (top oxide)-passivated DGAAS over 28 days, measured in ambient air at V_D = 1 V with the device stored in vacuum between measurements. (a) Transfer characteristics. (b) Peak current and peak voltage as a function of time. ΔI_{peak} and ΔV_{peak} denote the maximum changes relative to day 1.

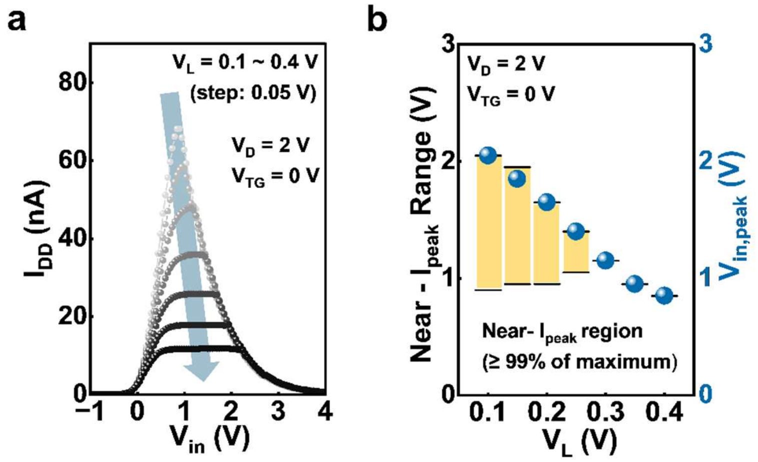

Figure S4. (a) AAS cell current as a function of V_{in} for V_L = 0.1–0.4 V, showing the constant-current plateau. (b) Near-I_{peak} range (≥ 99% of maximum) and V_{out} peak position as a function of V_L, confirming that the plateau ends at the V_{out} peak.

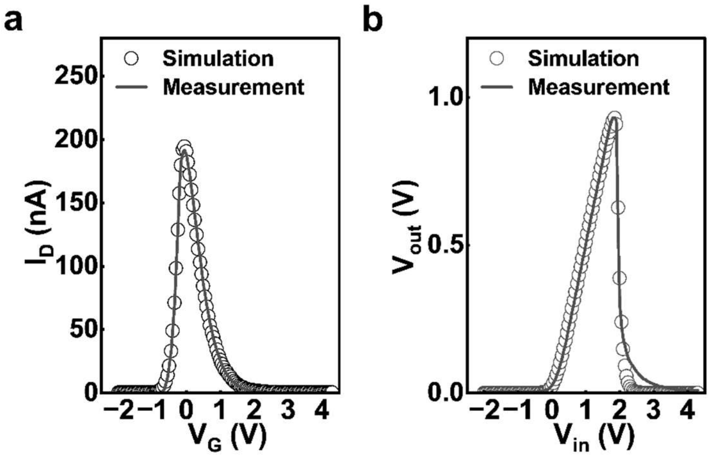

Figure S5. Compact-model fitting to the measured characteristics of (a) the DGAAS (I_D–V_G) and (b) the AAS cell (V_{in}–V_{out}).

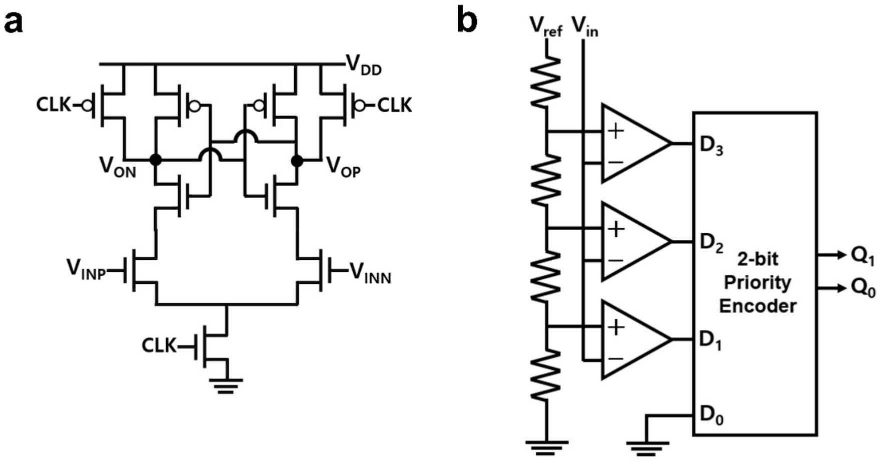

Figure S6. Baseline CMOS flash ADC. (a) Dynamic latch comparator. (b) Comparator array with a 2-bit priority encoder.
